# Illustrations of the Influence of Pregnancy*Read before the Association of the College of Physicians of Ireland.

**Published:** 1856-01

**Authors:** W. F. Montgomery

**Affiliations:** Professor of Midwifery to the King and Queen’s College of Physicians


*Illustrations of the Influence of Pregnancy in controlling or retarding the Development of certain Diseases.** By W. F. Montgomery, M. D. Professor of Midwifery to the King and Queen's College of Physicians.

On a former occasion, when treating of the condition of pregnancy, I took occasion to remark that it appeared from experience, that women who bear children, generally enjoy more even health, and are less disposed to disease than those who lead a life of celibacy, or, who, having married, remained unfruitful; so that Gardien seems to express no more than the truth when he says, "*Des qu'une femme est grosse les probabilites de sa vie augmentent;*" and this is what we ought, *a priori*, to expect, because child-bearing being the ordinance of an Alwise Providence, we should anticipate that the fulfillment of the duty thus ordained, would conduce to the welfare of those on whom it has been devolved.

It seems in conformity with such a view to believe, what indeed, I think, experience has taught us, that pregnancy acts in a great degree, as a protection against the reception of disease, and perhaps on the common principle, that during the continuance of one very active operation in the system, it is thereby rendered less liable to be invaded or acted on by another; thus it has been observed, that during epidemics of contagious diseases of different kinds, a much smaller proportion of pregnant women have been attacked than of others; but when attacked, they suffer severely: thus, when the cholera visited this country, the proportion of pregnant women who took the disease was very small, but all who caught it died, I believe, without almost a single exception. Gardien's experience led him to a similar conclusion; he says: "*Les femmes enceintes sont moin, exposees agagner les maladies contagieuses; mais lorsqu' elles ensont atteintess elles succombent plus promptement.*" I think, also, I have seen sufficient to satisfy me that pregnancy does, at least occasionally, exercise another kind of influence over disease in the system, namely, of preventing its

* Read before the Association of the College of Physicians of Ireland.

development during that state, although the infection may have been caught; as is proved by the disease showing itself immediately after delivery, as in the following cases:

Mrs. W., when in the ninth month of pregnancy, was much about her brother, who was dangerously ill of malignant scarlatina; she seemed to have escaped the danger completely; but the day after her delivery, she was covered with the disease, of which she died in a few days; between the time of her exposure to the infection and her delivery, there had intervened three weeks, during which she appeared to be quite well.

When Mrs. F. was in the eighth month of pregnancy, her husband had typhus fever; in which she assiduously attended him; after his recovery, she went to her father's house, some fifty miles from town, where she was delivered in due time, and immediately afterwards was seized with typhus fever, of which she died in eight days; between five and six weeks had elapsed between Mr. F.'s illness and her labor, and during that interval, she seemed in perfect health.

In the month of November, 1854, I attended a young lady in her first confinement; previous to which, she had both the lower extremities much enlarged by anasarca, but she appeared, in other respects, quite well, with one exception, which was that she had such *soreness* of the abdomen, she found a difficulty in laying on either side: and when I passed my hand over the abdomen, she complained that the pressure hurt her everywhere.

On the 12th she was confined, after a favorable labor, but the abdominal tenderness remained, and there was a peculiar doughy feel of the whole abdomen; next day, this was equally felt, but with little or no pain or fever, and a perfectly quiet pulse.

On the 14th I found the insteps of both feet, but particularly the left, covered with well developed erysipelas; her mother, who seemed very anxious about her, was present when I examined the feet, and on our reaching the drawing-room, said, "Doctor, isn't that very much like erysipelas?" I said, "Yes, certainly, there was no doubt about it." "Dear me, sir, do you think she could have taken it from her husband?" She then, for the first time, informed me, that some weeks before leaving home, to come to town for her confinement, her husband had a severe attack of erysipelas, during which she had assiduously nurse-tended him. Immediataly on the appearance of the erysipelas on the feet, the abdominal symptoms began to decline, and, after two or three days, ceased to exist. I cannot but believe that this lady caught the infection from her husband during her close attendance on him—that it remained in abeyance until gestation was over, and was then developed. She recovered well.

It is, I believe, a matter of common observation, that when women who have been laboring under certain forms of disease happen to conceive, the morbid affection previously existing is oftentimes either greatly mitigated, checked, or even altogether suspended for a time, as has been frequently observed in persons affected with phthisis; though I must add, that the influence of pregnancy in cases of phthisis is a question on which a variety of discordant opinions has been given by high authorities. Andral's conclusion, from his latest observations, is, "that in the great majority of cases the symptoms of phthisis are suspended, or at least remain stationary during the course of pregnancy." Louis says he is not "in a condition to determine whether pregnancy is, or is not capable of retarding the progress of phthisis," but he suggests that the fact might be, that several of the symptoms become somewhat more obscure during pregnancy, without any check being in reality given to the advance of the disease. My own experience would lead me to the conclusion, that if a woman, predisposed to phthisis, but in whom the disease has not actually become developed, prove pregnant, she is likely to be benefitted thereby; and I think I have seen life thus prolonged, for years, in several instances; but, on the other hand, if pregnancy takes place in a woman already actually in consumption, or if this disease supervene on pregnancy, the fatal issue is as likely to be accellerated as postponed, or, perhaps, even more so.—*Dublin Quarterly Jour. Med. Sci and Med. Exam.*

